# Coordination copolymerization monitoring of ethylene and alfa-olefins by in-line Raman spectroscopy†

**DOI:** 10.1039/d2ra05213j

**Published:** 2022-10-10

**Authors:** Víctor E. Comparán-Padilla, Maricela García-Zamora, Ramiro Infante-Martínez, José A. Díaz, Luis Villarreal-Cárdenas, María T. Rodríguez-Hernández, Odilia Pérez-Camacho

**Affiliations:** Departamento de Química Macromolecular y Nanomateriales, Centro de Investigación en Química Aplicada (CIQA) Blvd. Enrique Reyna H. 140, Col. San José de los Cerritos Saltillo 25294 Coahuila Mexico; Departamento de Procesos de Polimerización, Centro de Investigación en Química Aplicada (CIQA) Blvd. Enrique Reyna H. 140, Col. San José de los Cerritos Saltillo 25294 Coahuila Mexico; Laboratorio Central, Centro de Investigación en Química Aplicada (CIQA) Blvd. Enrique Reyna H. 140, Col. San José de los Cerritos Saltillo 25294 Coahuila Mexico

## Abstract

Copolymerizations of ethylene and alfa-olefins, using Ziegler–Natta or metallocene catalysts, testing two methods of co-monomer addition, through batch or dossing mode during the reactions, are reported in this work. Copolymerizations are monitored by in line Raman spectroscopy, comparing the effect of the kind of catalyst and the co-monomer addition modes on the chemical composition of the copolymers produced. The global co-monomer composition is determined by ^13^C NMR spectroscopy, compared with the monitoring by Raman spectroscopy along the reactions, where it is possible to define homogeneous or heterogeneous co-monomer distributions. Batch addition achieves higher incorporations of co-monomers, compared to dosed addition, where it is possible to determine the maximal co-monomer addition without affecting activities by transfer reactions. The incorporation mode of alfa-olefins in this type of reaction has been little reported, and until it is known, there is no rapid technique available to determine the uniformity of the co-monomer incorporations in real time. Copolymerization kinetics are also reported here and correlated to the addition method of the comonomers in both kinds of reactions. Homogeneous and heterogeneous co-monomer incorporations promoted by a single site catalyst (metallocene) or multisite system (Ziegler–Natta) is related to the homogeneous or heterogeneous co-monomer distributions detected by Raman spectroscopy, using each kind of catalytic system.

## Abstract 2

Copolymerizations of ethylene and alfa-olefins, using Ziegler–Natta or metallocene catalysts, testing two methods of co-monomer addition, through batch or dossing mode during the reactions, are reported in this work.

## Graphical abstract

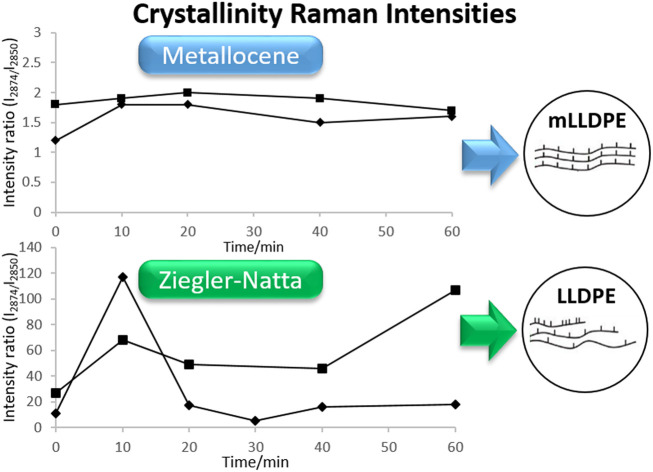

## 1. Introduction

Among the different types of polyethylenes, linear low density (LLDPE) is a special material due to its unique properties in casting and low sensitivity to degradation by shear stress.^1–3^ LLDPEs are copolymers obtained from the coordination polymerizations of ethylene and alpha-olefins such as 1-butene, 1-hexene, 1-octene and 1-decene, among others.^4–6^ The microstructure of these materials of linear chains with long or short branches allows partial crystallization, modifying the properties and also rheological behavior.^1,7,8^ LLDPE shows greater tensile strength, and resistance to impact and puncture compared to low density polyethylene (LDPE), being a widely used material in advanced applications and films of high resistance.^9,10^

LLDPE can be obtained with both metallocenes, or Ziegler–Natta catalysts, however, copolymers obtained with Ziegler–Natta catalysts have broad molecular weight distribution (*Đ* > 5), and one of its main drawbacks is the non-homogeneous incorporation of the comonomer in the polymer chains.^4–6^ On the other hand, copolymers obtained with metallocenes (mLLDPE), being “Single Site Catalysts” (SSC), usually produce copolymers with narrow molecular weight distributions (*Đ* < 5) and a more homogeneous comonomer incorporation.^11,12^ Due to the homogenous microstructure, mLLDPE shows improved physical and mechanical properties compared to LLDPE synthesized with Ziegler–Natta catalytic systems.^13^ The reaction parameters in coordination copolymerizations of ethylene and alfa-olefins have a great effect on the chemical composition, and other features that affect end properties of the copolymers. For instance, the mode of comonomer addition in the reactions, can affect the incorporation degree of this in the LLDPE's, varying their chemical composition and therefore their morphology and crystallinity. Until our knowledge, there are no reports in the literature about the study of addition methods of comonomers in coordination copolymerizations, and other reports only describe the process adding the total amount of comonomer since the beginning of the reaction (batch mode). Concerning to the chemical characterization of LLDPE's obtained with both metallocenes or Ziegler–Natta catalysts, ^13^C NMR is a precise quantitative technique which is usually utilized to determine the percent mol of incorporation of alfa-olefins in LLDPE's, however does not give information about the type of homogeneous or heterogeneous distribution of the comonomer in the materials. Homogeneous comonomer incorporations like the one obtained in copolymerizations using single site systems (metallocene catalysts) are related to the higher crystallinity degree in the polymers. As for other chemical characterization techniques, since 1996 Tashiro and co-workers utilized Raman spectroscopy for linear polyethylene analysis, confirming the spectral patterns of disordered short segments and crystalline phases in ethylene homopolymers, correlating structural changes with crystalline phases of PE through different techniques and Raman spectra.^14^ Later, the reports of Nikolaeva and Sagitova^15,16^ demonstrated the regularity of vibrational modes in Raman spectroscopy, at high wave number spectral regions (1000–1600 cm^−1^ and 2750–3200 cm^−1^) for several polyolefins and random ethylene copolymers. In the last report, they confirmed that the peak positions and intensities depend on the length and content of sequences of ordered segments related to the degree of crystallinity, or the content of different conformers corresponding to the amorphous phase in polyolefins. Besides, they also confirmed the Raman peaks assignation corresponding to different chemical bonds in polyethylenes and ethylene copolymers.^14–16^

In other report concerning to linear polyethylenes characterizations by Raman spectroscopy, Deshpande^17^ used a Raman fiber optic probe introduced in the polymerization reaction vessel, obtaining *in situ* data about the consumption of the comonomers concentration. A predictive chemometric model through mass balances was utilized in this process, to obtain quantitative results of the comonomer depletion during the copolymerization reactions. According to the results of Deshpande,^17^ Rattiste,^18^ and other reports in slurry copolymerizations,^19^ the *in situ* Raman probe provided important information, however the spectral region analysed in this study (544 to 1720 cm^−1^) was restricted to higher wave number regions, limiting the analysis of conformational arrangements related to crystalline and amorphous phases in the polyolefins.

From the above literature reports focused to the study of linear polyethylenes and ethylene–alfa olefin copolymers, it is evident that Raman spectroscopy is a sensible technique to analyse crystalline and amorphous polymer phases, which can be related to the homogeneous and non-homogeneous comonomer incorporations in the synthesis of LLDPE.

In the present work, two kind of catalytic systems (metallocene and Ziegler–Natta) were studied in ethylene and alfa-olefins copolymerizations, where the incorporation mode of the comonomer was also compared through continuous flow along the copolymerizations, or by batch mode (comonomer initial charge). Copolymerizations were monitored by in line Raman spectroscopy, analysing amorphous phases formation, which gave information about the chemical composition of the copolymers. Monitoring was carried out *in situ*, using a special probe immersed into the polymerization reactor and connected to the Raman spectrometer, comparing to NMR analyses, GPC characterizations and thermal properties of the copolymers obtained.

## 2. Experimental section

### 2.1 Materials

*n*-Hexane, 1-hexene, 1-octene, modified methyl-aluminoxane MMAO-12 (10% weight in toluene), tri-isobutilauminio TIBA (25% weight in toluene) and bis(cyclopentadienyl)zirconium dichloride 99% (Cp_2_ZrCl_2_) were obtained from Sigma-Aldrich. HD-46 Ziegler–Natta commercial catalyst was obtained from Albemarle Chemical. Ethylene 98.98% C.P. grade and N_2_ UAP from Infra.

### 2.2 Instrumentation

Copolymerizations were carried out in a 1 L glass Parr reactor, provided with lines for vacuum and N_2_ or ethylene pressurization. The vessel reactor jacket was connected to a heat exchanger thermal circulator. The ethylene flow was measured by a mass flow controller CFG Aalborg. A high-pressure piston pump SSI Series III from Scientific Systems was employed for the comonomer dosification. Hardware cDAQ-9178 and software LabVIEW, both from National Instruments were used for data acquisition and supervisory control functions. In-line Raman spectrometry was performed by a Smart Raman Spectrometer DXR2 from Thermo Scientific provided with a 5 m long optic fiber cable with a laser of 785 nm.

To determine the molecular weight of the polymers, an Alliance GPC 2000 Gel Permeation Chromatograph was used at 140 °C at a flow rate of 1 mL min^−1^, and an injection volume of 219.5 μL of 1,2,4-tri-chlorobenzene. Narrow molecular weight distribution polystyrene standards from 1950 to 3 250 000 g mol^−1^, were used for GPC calibration. The ^13^C NMR spectra were obtained on a Bruker Ultrashield 500 MHz Plus spectrometer using ethylene tetrachloride (C_2_Cl_4_) and a capillary with deuterated toluene as external reference. The thermal analysis tests of the polymers were performed in a DSC TA instrument 2920 modulated. The samples (8–10 mg) were subjected to three heating–cooling ramps from 25 °C to 200 °C at a temperature rate of 10 °C min^−1^.

### 2.3 Polymerization and copolymerization reactions of ethylene and 1-hexene or 1-octene

Prior to each polymerization, the reactor vessel was heated to 90 °C, under vacuum for 1 hour. Subsequently, the reactor was charged with 300 mL of dry hexane, and in the case of the batch copolymerization reactions, the total amount of the comonomer was added. Three vacuum-nitrogen cycles were made to degas the system, and then 2 mL of TIBA was added as “scavenger” and the system was pressurized to 42 psi of ethylene. Then the mixture was heated to 70 °C to remove impurities, for 30 minutes, with agitation of 350 rpm. Meanwhile, in a Schlenk flask, the corresponding amount of metallocene catalyst (0.01 g) was weighted, dissolved in dry toluene (5.6 mL) and activated with 8.8 mL of MMAO-12 corresponding to a ratio of Al/Zr = 250. The mixture of the Schlenk flask was stirred for five minutes and added to the polymerization reactor using a syringe with valve. After injecting the catalyst, the ethylene valve was opened, raising the temperature to 70 °C, maintaining a constant pressure of 42 psi and agitation of 350 rpm. As for the Ziegler–Natta commercial catalyst, after “scavenger” step, 0.4 mL of activator (TEA), corresponding to a ratio of Al/Ti = 10, was diluted in dry hexane (5 mL) and added to the polymerization reactor using a syringe with valve. Then, 0.5 mL of the catalyst solution (20 wt% in hexanes) was put in a Schlenk flask and dissolved in dry hexane (5 mL) and added to the polymerization reactor using a syringe with valve, finally the ethylene valve was opened.

The ethylene consumption was registered during one hour of reaction, after this time the ethylene valve was closed, the agitation was suspended, and the temperature was set at 25 °C. Subsequently, 20 mL of acidified methanol (10% hydrochloric acid) were added to the reactor for deactivating the catalyst and the aluminum compounds, stirring for 30 minutes. Then the polymer was washed three times with 200 mL of methanol, stirring for 15 minutes, filtering the polymer in each wash, which finally was dried for 6 hours at 60 °C.

For copolymerizations of ethylene and 1-hexene or 1-octene with continuous comonomer addition, the comonomer, previously distilled and degassed was transferred to the dosing system under nitrogen atmosphere. Subsequently, the conduits were purged with comonomer solution, and connected to the polymerization reactor. The pump was programmed for a rate of addition of 0.5 mL min^−1^, whose flow started after the introduction of the catalyst into the reactor. The detection probe of the Raman equipment was introduced into the polymerization slurry and recorded in real time. The addition/dosage system was prepared before the thermal conditioning of the reactor.

## 3. Results and discussion

Copolymerizations of ethylene and 1-hexene or 1-octene were performed using two different catalytic systems (metallocene or Ziegler–Natta), and two methods of co-monomer addition (continues or batch mode), monitoring the reactions by Raman spectroscopy through the use of a special probe immersed into the polymerization reactor, and connected to the Raman equipment. Classical zirconocene Cp_2_ZrCl_2_ at concentrations of 6–10 × 10^−5^ mol L^−1^, was activated at low Al/Zr ratios established at 250 equivalents (Al/Zr = 250) in order to avoid large excesses of inorganic compounds that could generate interferences in the Raman detection probe. It is worth mentioning that other polymerization conditions like ethylene pressure and solvent, were stablished according to copolymerizations carried out in previous reports.^20^ As for the Ziegler–Natta catalyst, a commercial fifth generation system from Grace Co., based on Ti, was utilized at 6.9 × 10^−4^ mol L^−1^, which was activated with triethylaluminium (TEA) at Al/Ti ratios of 10, according to the usually conditions utilized for these systems.^11^

In order to establish the initial amount of comonomer added batchwise or gradually dosed during the copolymerization reactions, preliminary reactions of ethylene homopolymerizations were carried out with both systems (metallocene or Ziegler–Natta). The results of the homopolymerization and copolymerization reactions with the metallocene and the Ziegler–Natta catalysts are shown in Table 1 and 2, respectively. The “blank” experiment (Table 1) corresponding to the homopolymerization of ethylene using the metallocene system, showed an activity of ∼1021 Kg PE mol^−1^ Zr h^−1^. Based on the amount of PE obtained, it was determined to add 10 g of co-monomer (1-hexene or 1-octene) in the copolymerizations, corresponding to 17 wt% with respect to PE produced. However, it should be noted that in this type of polymerizations by coordination, as in the most of the addition polymerizations, the incorporation of the comonomer in the copolymer obtained is generally less or different than the initial addition, with an amount of unreacted monomer left in the copolymerization systems. Experiments 1 and 2 correspond to ethylene and 1-hexene copolymerizations of batch and dosed comonomer addition, respectively. In experiments 1 and 3, 10 g of co-monomer were added at the beginning of the reaction, as detailed in the experimental part, and for experiments 2 and 4, the addition of the comonomer was dosed at 0.33 or 0.35 mL min^−1^, respectively along one hour of reaction. Catalytic activities of the batch and dosed copolymerizations are compared in Table 1 with the corresponding activity of ethylene homopolymerization (“blank”), as well as the molar masses and co-monomer incorporations, of the polyethylene and copolymers obtained.

**Table 1:** Homopolymerization and copolymerization reactions of ethylene and comonomers (1-hexene or 1-octene) using two comonomer addition methods and metallocene catalyst^a^

Exp.	Com	Com initial (g)	*A* Kg PE (mol Zr h)^−1^	*M*_n_ (g mol^−1^)	*Đ*	% mol com
Blank	—	—	1021	18 360	3.3	—
1	1-Hex	10	720	5434	3.1	0.9
2*	1-Hex	0.33	1015	16 370	3.5	1.0
3	1-Oct	10	1043	6750	2.5	1.5
4*	1-Oct	0.35	1431	17 238	5.0	0.5

a *A* = Activity in Kg PE (mol Zr h)^−1^; *Đ* = *M*_w_/*M*_n_; [Zr] = 6–10 × 10^−5^, Al/Zr = 250, *v* = 350 mL hexane, 42 psi or 2.9 bar of ethylene, *T* = 70 °C, 350 rpm. Exp 1, initial charge of 1-hexene (10 g); exp 2* continuous dosage of 1-hexene at 0.33 mL min^−1^ (10 g, along the reaction); exp 3, initial charge of 1-octene (10 g); exp 4* continuous dosage of 1-octene 0.35 mL min^−1^ (10 g, along the reaction); mol% of co-monomers determined by ^13^C NMR.

**Table 2:** Homopolymerization and copolymerization reactions of ethylene and comonomers (1-hexene or 1-octene) by two comonomer addition methods using Ziegler–Natta catalyst^a^

Exp.	Com	Com initial (g)	*A* Kg PE (mol Zr h)^−1^	*M*_n_ (g mol^−1^)	*Đ*	% com
Blank	—	—	206	7164	32	—
5	1-Hex	10	182	9336	21	5
6*	1-Hex	0.5	193	12 554	32	1.5
7	1-Oct	10	206	13 590	17	3.5
8*	1-Oct	0.5	199	12 706	18	1

a *A* = Activity in Kg PE (mol Zr h)^−1^; *Đ* = *M*_w_/*M*_n_; exp 6* continuous dosage of 1-hexene at 0.33 mL min^−1^ (10 g, along the reaction); exp 5, initial charge of 1-hexene (10 g); exp 7 initial charge of 1-octene (10 g); exp 8* continuous dosage of 1-octene at 0.33 mL min^−1^ (10 g, along the reaction); mol% of co-monomers determined by ^13^C NMR.

As has been reported in other copolymerizations, a negative effect of 1-hexene in the copolymerization reaction was observed, since catalytic activity decreased by 30% with respect to the blank without comonomer (exp 1, Table 1).^21,22^ Ethylene and 1-hexene copolymer, also presented a decrease in molar mass, compared to the blank, indicating that the initial excess of 1-hexene led to chain transfer reactions to the comonomer, forming less reactive species. Unlike experiment 2*, where a gradual and slow addition of the comonomer was done along the reaction, activities remained unchanged, and the molecular weight decreased just a 10% compared to the molecular weight of the homopolymer (blank). The incorporation of 1-hexene showed a very similarly low value around 1% in both copolymerizations, determined by ^13^C NMR. However, in the case of the copolymerization with gradual addition of 1-hexene, the comonomer had no negative effect on the activity, neither on the molecular weight of the polymer, probably due to the low initial concentration, avoiding the chain transfer reactions to the comonomer.

As for 1-octene copolymerizations (exp 3 and 4*, Table 1), a positive effect on the activity was observed, even by batch addition method, where an increase of 35% of copolymer production was obtained and a higher incorporation of 1.5 mol% was detected by ^13^C NMR, however the molecular weight decreased, compared to the blank more than 50% (compare exp. 1 and 3, Table 1).

**Fig. 1:**
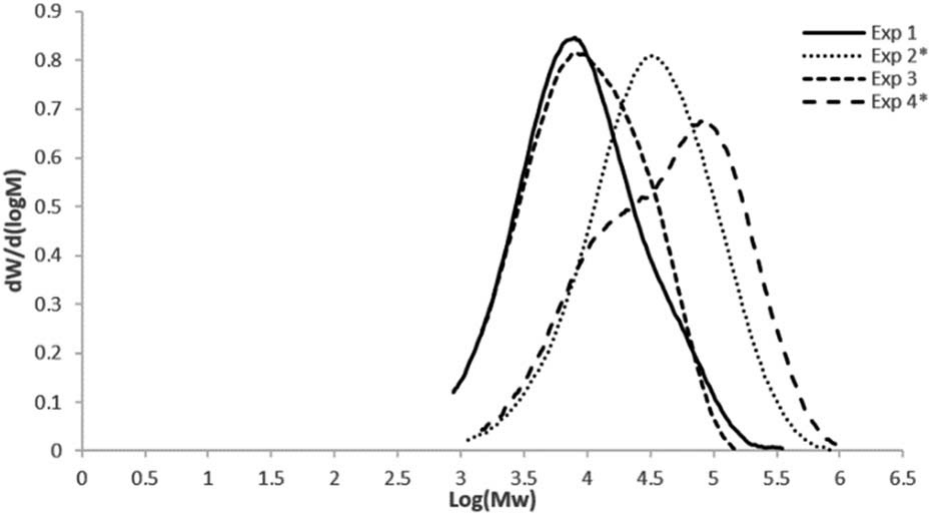
Molar mass distributions of the 1-hexene and 1-octene ethylene copolymers obtained with zirconocene catalyst. Uncomplete curves are due to low *M*_w_ copolymers obtained in the reactions, which are out of the standards values (1950 to 3 250 000 g mol^−1^) used for GPC calibration.

Fig. 1 shows the molar mass distributions of the different 1-hexene and 1-octene–ethylene copolymers obtained with metallocene system, showing monomodal curves, with exception of the Exp. 4 (dosed addition of 1-octene), with dispersities between 3 and 5, characteristic of single site catalysts (SSC).

Table 2 displays homopolymerization (blank) and copolymerization results of ethylene and 1-hexene or 1-octene using the commercial Ziegler–Natta catalyst. Similar to those comonomer incorporation results, showed in Table 1, for metallocene catalyst, the comonomer additions by lots (exp. 5 and 7) for 1-hexene and 1-octene, respectively, presented the highest incorporation grades, determined by ^13^C NMR. However, with the Ziegler–Natta system, the incorporation of 1-hexene under these polymerization conditions, reached higher percent (5 mol%, exp. 5) than with 1-octene (3.5 mol%, exp. 7), and also higher than those corresponding to metallocene polymerizations (Table 1). On the other hand, a positive comonomer effect was observed for all of these copolymerizations showed in Table 2, compared to the activity of the blank, and although copolymers obtained with Ziegler–Natta systems could present high comonomer contents, homogeneous compositions are difficult to achieve, since this kind of catalysts contain heterogeneous active sites. It is well known that different active sites, in Ziegler–Natta systems lead to non-homogeneous copolymers, because most of the comonomer is incorporated into short polymeric chains.^23^ Multiple active sites also correspond to molecular weight data observed in Table 2, where an increase in the MW of the copolymers compared to the blank, indicate the positive co-monomer effect, and the wide molar masses distributions, correspond to the heterogeneous catalytic system.

**Fig. 2:**
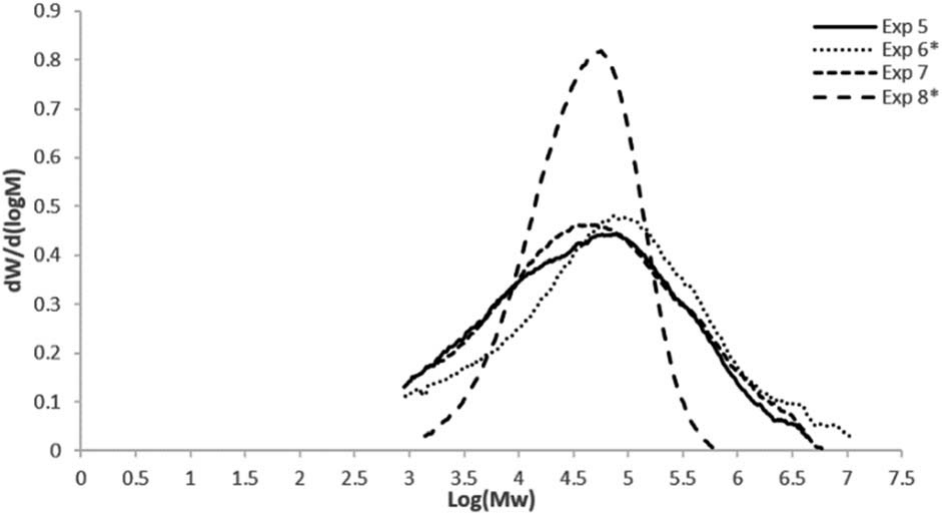
Molar mass distributions of the 1-hexene and 1-octene ethylene copolymers obtained with the commercial Ziegler–Natta catalyst. Uncomplete curves are due to low *M*_w_ copolymers obtained in the reactions, which are out of the standards values (1950 to 3 250 000 g mol^−1^) used for GPC calibration.

Fig. 2 displays the molar mass distributions for the copolymers obtained with the heterogeneous catalytic system (commercial Ziegler–Natta catalyst), where as expected, much wider distributions were obtained, compared to the SSC.

Unlike metallocene copolymerizations, with single active sites, where the comonomer is homogeneously distributed in all the polymeric chains. Homogeneous comonomer distributions have significant impact on the physical properties and behavior of the materials, therefore is important to have some kind of evidence about the microstructural characteristics in this kind of copolymerization reactions. Chemical composition distribution (CCD) in polyolefin copolymers, has been widely studied by thermal fractionation methods, like CEF (crystallization elution fractionation) a high-resolution technique, which can provide precise and quantitative information about the CCD, however for industrial uses, this is not a practical method, due to their long measurement times and the use of high-temperature solvents.^23^

In the present study, the comonomer incorporation was verified by Raman spectroscopy, monitoring in line by introducing a Raman probe into the slurry polymerization reactor, obtaining rapid qualitative information related to CCD in the copolymer's formation. The assignments of the Raman lines obtained in the analyses of the copolymers produced, are based in the studies reported by Nikolaeva and co-workers and other authors,^14–16^ about ethylene copolymers and high alfa-olefins like 1-hexene and 1-octene, at several comonomers contents. Through Raman technique has been possible to obtain information about the crystalline and amorphous phases in polyolefins produced by the orientational order of the macromolecules in the materials. Since Raman spectroscopy is a very sensitive technique to isotropic and deformed states, the incorporation of high comonomers like 1-hexene or 1-octene, even at low concentrations in polyolefins can be verified, where also can be simultaneously determined the maximal number of structural characteristics in the copolymers. Nikolaeva and co-workers^15,16^ assigned the main Raman lines in ethylene copolymers based on the regularity modes, where the peak positions and intensities depend on the length and content of CH_2_-chain segments (conformationally ordered). The regularity modes correspond to vibrations of *trans*- or *gauche*-conformers that decrease, or move its position, as the incorporation of the comonomer increases in the PE chains. They compared the intensities and peak positions of the regularity modes, to those of the corresponding low-density polyethylene and ethylene copolymers at different comonomer contents and temperatures, observing that crystalline and amorphous phase compositions are affected. Thus, the main Raman active bands for different organic compounds that contain polymethylene chains as well as for ethylene copolymers, could be assigned to the most common chemical bonds. The stretching vibrations of C–C bonds have been assigned at 1062 and 1130 cm^−1^ for asymmetric (*ν*_as_(C–C)) and symmetric (*ν*_s_(C–C)), respectively. At 1170, 1295 and 1415 cm^−1^ the peaks were assigned to the rocking (*r*(CH_2_)), twisting (*τ*(CH_2_)) and wagging (*ω*(CH_2_)) vibrations of the methylene groups, respectively, where all of these bands are of medium intensity. As for C–H bonds of CH_2_ groups, the bands at 2850 and 2883 cm^−1^ were assigned for the symmetric (*ν*_s_(CH_2_)) and the asymmetric (*ν*_as_(CH_2_)) vibrations respectively. In addition, it was also confirmed that in ethylene copolymers, crystalline phase is formed by all *trans*-conformers, while the non-crystalline or amorphous phase is produced by macromolecules with a significant amount of *gauche*-conformers.^15^ On the other hand, based on Raman spectra of *n*-alkanes, Zerbi *et al.* identified a group of lines assigned as C–C stretching of the end groups of CH_2_ conformers from 800 to 900 cm^−1^ (end *trans*–*trans* at 890 cm^−1^, end *trans*–*gauche* at 870 cm^−1^ and end *gauche*–*trans* at 840 cm^−1^) generally observed in Raman spectra of conformationally disordered chains.^24^

Based on the reported assignments for Raman spectra of ethylene copolymers, in the present work, the main Raman bands were detected on line during the copolymerization reactions, even at low comonomer incorporations, and some differences could be observed for each single (metallocene) or multisite (Ziegler–Natta) catalytic system. The analyses were monitored along one hour of reaction, where initially, the solvent signals were the only visible bands (hexanes), however as the polymer formation advanced, the polyethylene and ethylene copolymer bands were also observed. Variations in the strongest vibrations on the region of 2830 to 3000 cm^−1^ corresponding to C–H bonds of methylene groups were detected, since are the predominant bonds in the material. Even at low contents of co-monomer incorporations (0.5 to 1.5 mol%) a comparison in the bands of 2850 cm^−1^ assigned to the *trans*-conformers of the crystalline phase, related to the intensities of the bands at 2876 cm^−1^ corresponding to the *gauche*-conformer segments, of the amorphous phase in the material, gave a qualitative evidence of the comonomers incorporation in polyethylenes. An increase in the ratio between the intensities of 2876 and 2850 cm^−1^ bands, correspond to amorphous phase increases, related to the content of comonomer in the polyethylenes. Even variations in the intensity ratios gave some evidence of the uniformity of the copolymers during its formation.

**Fig. 3:**
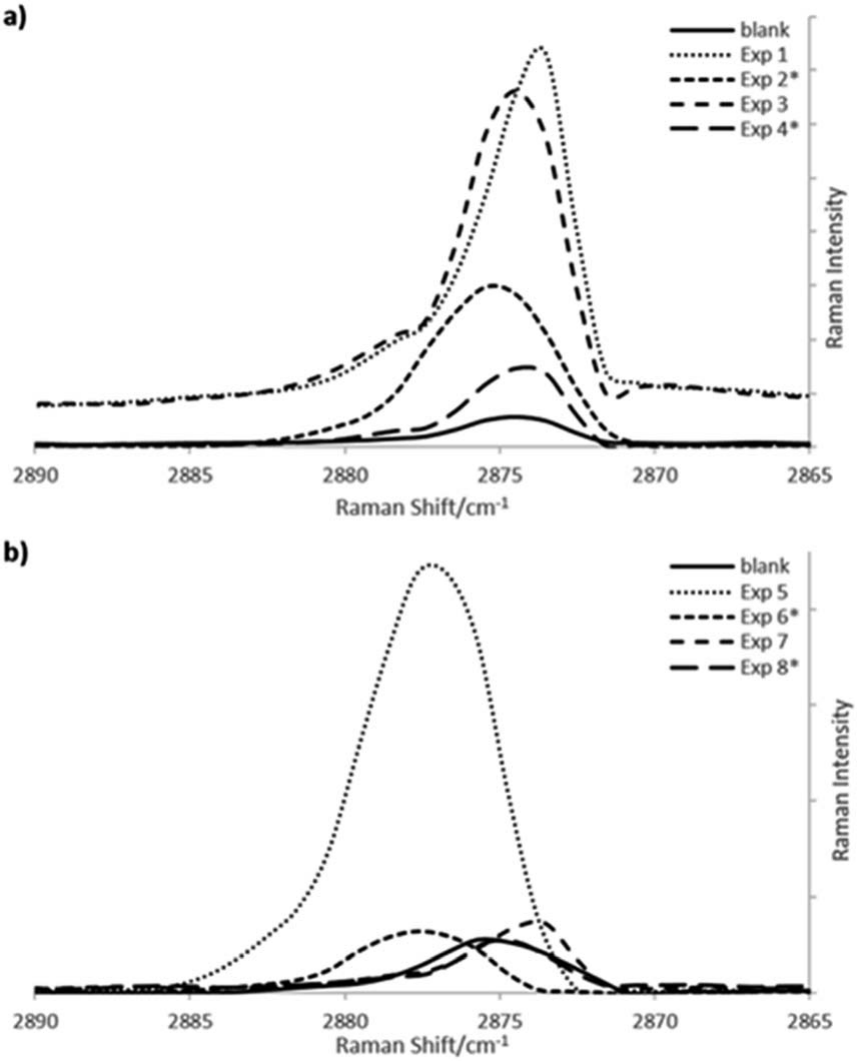
Raman spectra online after one hour of copolymerization of ethylene and 1-hexene or 1-octene (a) using the Cp_2_ZrCl_2_/MMAO-12 system (b) with Ziegler–Natta catalyst, in “batch” or dossing comonomer addition mode.

Fig. 3(a) and (b) compares representative Raman spectra of the C–H region, of the different copolymerization reactions carried out by lots or dossing of 1-hexene or 1-octene, along one hour of reaction, for both metallocene and Ziegler–Natta systems, respectively. Raman curves show high intensity signals at 2876 cm^−1^ corresponding to *gauche*-conformer segments for experiments 1 and 3 (Table 1) and experiments 5 and 7 (Table 2) due to higher amount of amorphous phase caused by the higher comonomer incorporations.

Intensities of 2876 cm^−1^ bands, corresponding to *gauche*-conformer vibrations, were normalized to the intensity of the 2850 cm^−1^ bands from the *trans*-conformer segments related to the crystalline phases. A decrease of the ratios (*I* = *I*_2876_/*I*_2850_) correspond to lower amounts of crystalline phase, having as contra part a higher concentration of amorphous phase from the incorporations of comonomer into the polyethylene chains. A small increase in the intensity ratios of *gauche*-conformer segments could be observed for the polyethylenes, what agree with the comonomer incorporation determined by NMR, around 1 mol%, where the highest incorporation obtained for exp. 3 adding 1-octene by lots (Table 1), shows the highest intensity ratio in the *gauche*-conformer Raman band.

If one compares Raman bands around 2876 cm^−1^ region from *gauche*-conformer segments of metallocene copolymerizations (Fig. 3a) with Ziegler–Natta reactions (Fig. 3b), more consistent shifts are observed for the single site system, in spite that the incorporation of co-monomers are lower than in the multisite system.

**Fig. 4:**
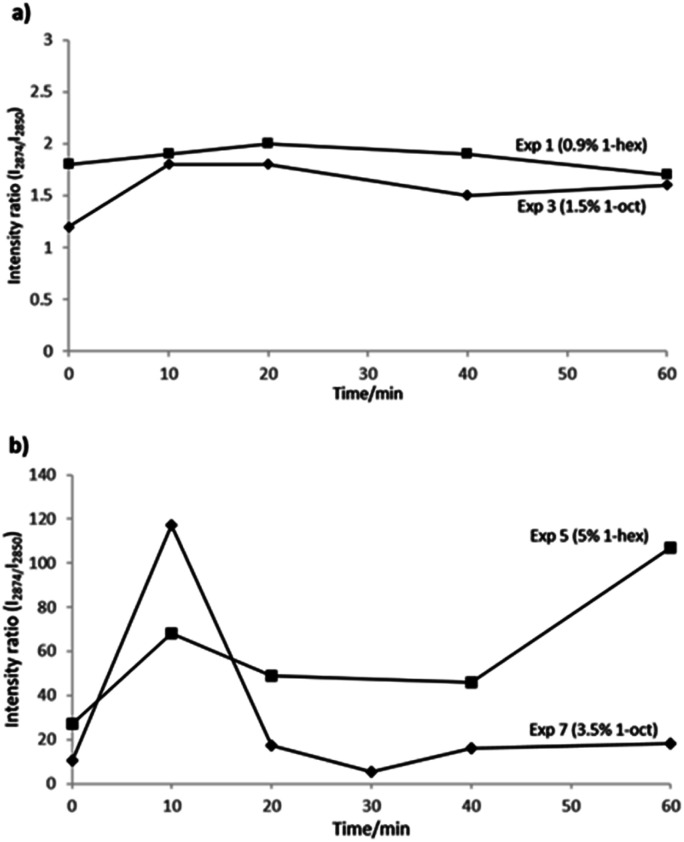
Intensity ratios of (*I* = *I*_2876_/*I*_2850_) obtained from the Raman spectra in real time, during one hour of copolymerization of ethylene and 1-hexene or 1-octene (a) using the Cp_2_ZrCl_2_/MMAO-12 system (b) with Ziegler–Natta catalyst, in “batch” comonomer addition mode.

Intensity ratios of the amorphous bands are compared in Fig. 4, for the copolymerization reactions carried out by batch comonomer addition mode, and using metallocene or Ziegler–Natta catalysts (Fig. 2a and b, respectively), in both cases for the copolymers with the highest incorporations.

Higher copolymer incorporation grades were obtained with Ziegler–Natta system, but larger variations of the intensity ratios were observed in the values plotted in Fig. 4b every ten minutes along the copolymerization reactions. As for metallocene copolymerizations, very low variations in intensity ratios, along the reactions can be observed in Fig. 4a. The last observation was expected, according to the homogeneous or heterogeneous kind of copolymerizations, obtained by single site or multisite catalytic systems, respectively.

Marked differences in the ethylene consumption of kinetic curves of each catalytic system are also noticed due to the active sites of each homogeneous or heterogeneous copolymerization system (Fig. S1, ESI†), which agree with the last Raman spectroscopic results.

**Fig. 5:**
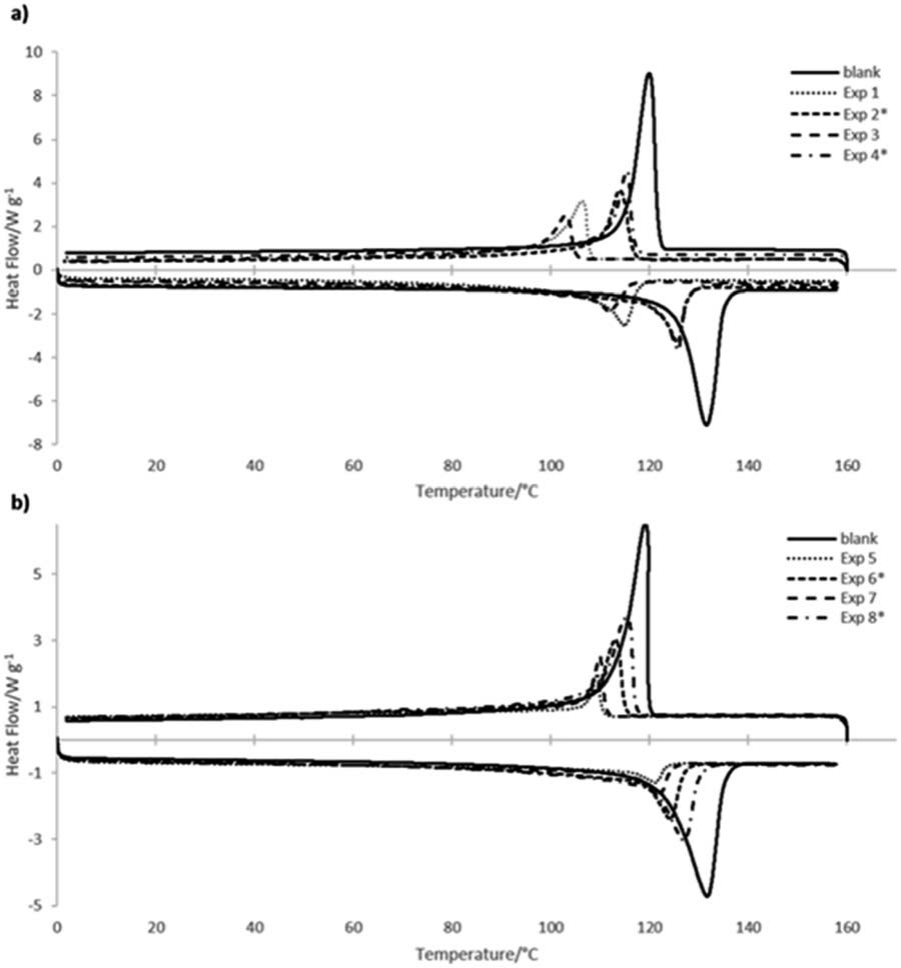
DSC thermograms of the copolymers obtained with (a) metallocene and (b) Ziegler–Natta catalysts, compared in both cases with the homopolymers (HDPE).

Polymerization kinetics of the metallocene and Ziegler–Natta systems, are compared in Fig. S1 in the ESI,† where the plots correspond to the consumption of ethylene *versus* reaction time, comparing the blank (homopolymerization) and copolymerizations in batch and with continuous addition of 1-hexene or 1-octene, respectively. As for the kind of comonomer addition, the copolymerizations carried out by dossing mode presented kinetic curves very similar to the blank polymerization without comonomers. Moreover, thermal characteristics of the obtained copolymers correspond to the homogeneous or non-homogeneous incorporation type in the copolymers generated by each catalytic system, also related to the observed in the Raman spectra. Fig. 5 shows the corresponding DSC curves, where in spite of the fact that copolymers obtained in the Ziegler–Natta polymerizations incorporated higher amounts of co-monomers (Fig. 5b), the copolymers obtained with metallocenes showed lower melting and crystallization temperatures in their curves (Fig. 5a), comparing in both cases with the homopolymers.

Thermal stability of the copolymers was probed through the thermogravimetrical analysis (TGA) of LLDPE with highest comonomer incorporations, obtained with metallocene or Ziegler–Natta catalysts. TGA analyses displayed in Fig. S2 in the ESI,† show high stability of the copolymers, until 400 °C, as expected for linear polyethylenes.

According to the Raman spectra obtained along the copolymerization reactions in real time, this characterization technique gives useful evidences about the homogeneity of the obtained copolymers, measuring the intensity of the amorphous phase. Quantification of the co-monomer incorporation is usually determined by NMR, however, at low co-monomer contents (<1%), ^13^C NMR spectra are difficult to obtain, and neither information about the co-monomer distribution can be detected by NMR techniques. On the other hand, is well known that homogeneous co-monomer incorporations generate materials of improved physical and mechanical properties, even at low contents of comonomers like 1-hexene or 1-octene. Therefore, the good performance of linear low density polyethylenes, also depends on the co-monomer distribution along the polymer chains, what can be easily verified by *in situ* Raman spectroscopy.

## 4. Conclusions

Coordination copolymerizations of ethylene and alfa-olefins (1-hexene and 1-octene), were studied using two different catalytic systems (metallocene and commercial Ziegler–Natta catalysts) where the effect of the addition method of the co-monomer, batch or continuous mode was monitored in line by Raman spectroscopy. The amorphous phase could be detected during the formation of the copolymers, using a Raman probe, and the uniformity of amorphous or crystalline bands could be observed even at low comonomer contents. Global co-monomer incorporations in the copolymers were determined by NMR, but more details about the chemical composition and homogeneity of the materials could be probed through *in situ* Raman analyses, during the reactions. Comparison between the addition methods of the co-monomers into the reactions gave evidence that the batch mode conduces to higher incorporation percent of both comonomers (1-hexene and 1-octene). Besides, could be determined that homogeneity of the co-monomer distribution is owed to the catalytic systems, and not to the continuous or batch mode addition, where the difference between metallocene and Ziegler–Natta, as single or multisite catalysts agree with the patron of broad or fine bands observed in the Raman spectra along the copolymerizations. From the results obtained in the present work, we conclude that *in situ* Raman spectroscopy gives precise information about co-monomer distributions, that makes complete the NMR quantification techniques for the chemical characterization of linear low density polyethylenes (LLDPE).

## Author contributions

Víctor E. Comparán-Padilla (methodology and investigation, figures); Maricela García-Zamora (methodology and data curation); Ramiro Infante-Martínez (conceptualization, funding and acquisition); José A. Díaz (methodology, resources); Luis Villarreal-Cárdenas (data curation, reactor instrumentation); María T. Rodríguez-Hernández (methodology, characterization analyses); Odilia Pérez-Camacho (conceptualization, funding acquisition writing original draft).

## Conflicts of interest

There are no conflicts to declare.

## Supplementary Material

RA-012-D2RA05213J-s001
