# Supplementary material for: Coordination copolymerization monitoring of ethylene and alfa-olefins by in-line Raman spectroscopy
Source: RSC Adv. 2022 Oct 10;12(44):28712–9. doi: 10.1039/d2ra05213j (PMC9549569; doi:10.1039/d2ra05213j)
Supplement: RA-012-D2RA05213J-s001 [file RA-012-D2RA05213J-s001.pdf]

Electronic Supplementary Material (ESI) for RSC Advances.
This journal is © The Royal Society of Chemistry 2022

Electronic Supplementary Information

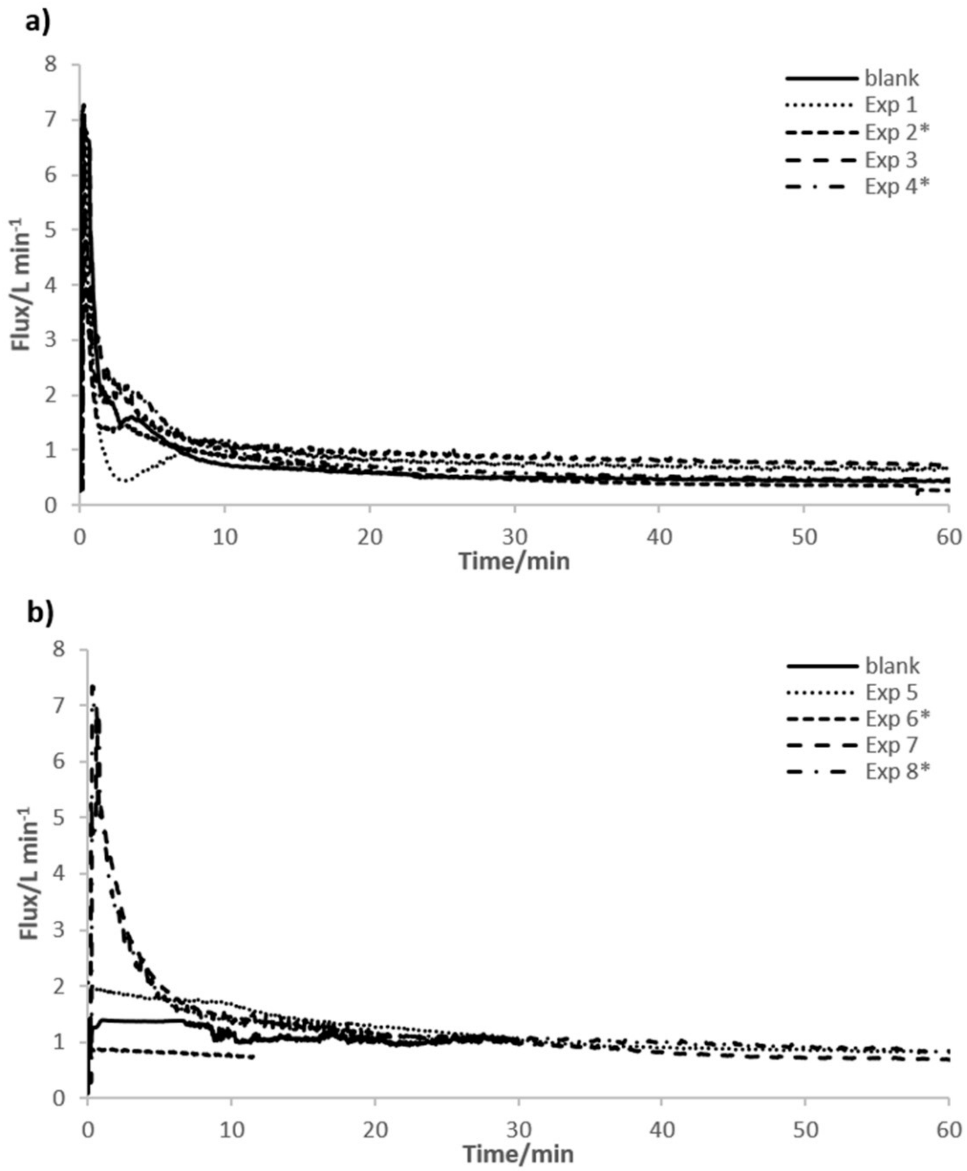

Figure S1. Polymerization profiles using a) metallocene and b) Ziegler-Natta catalyst

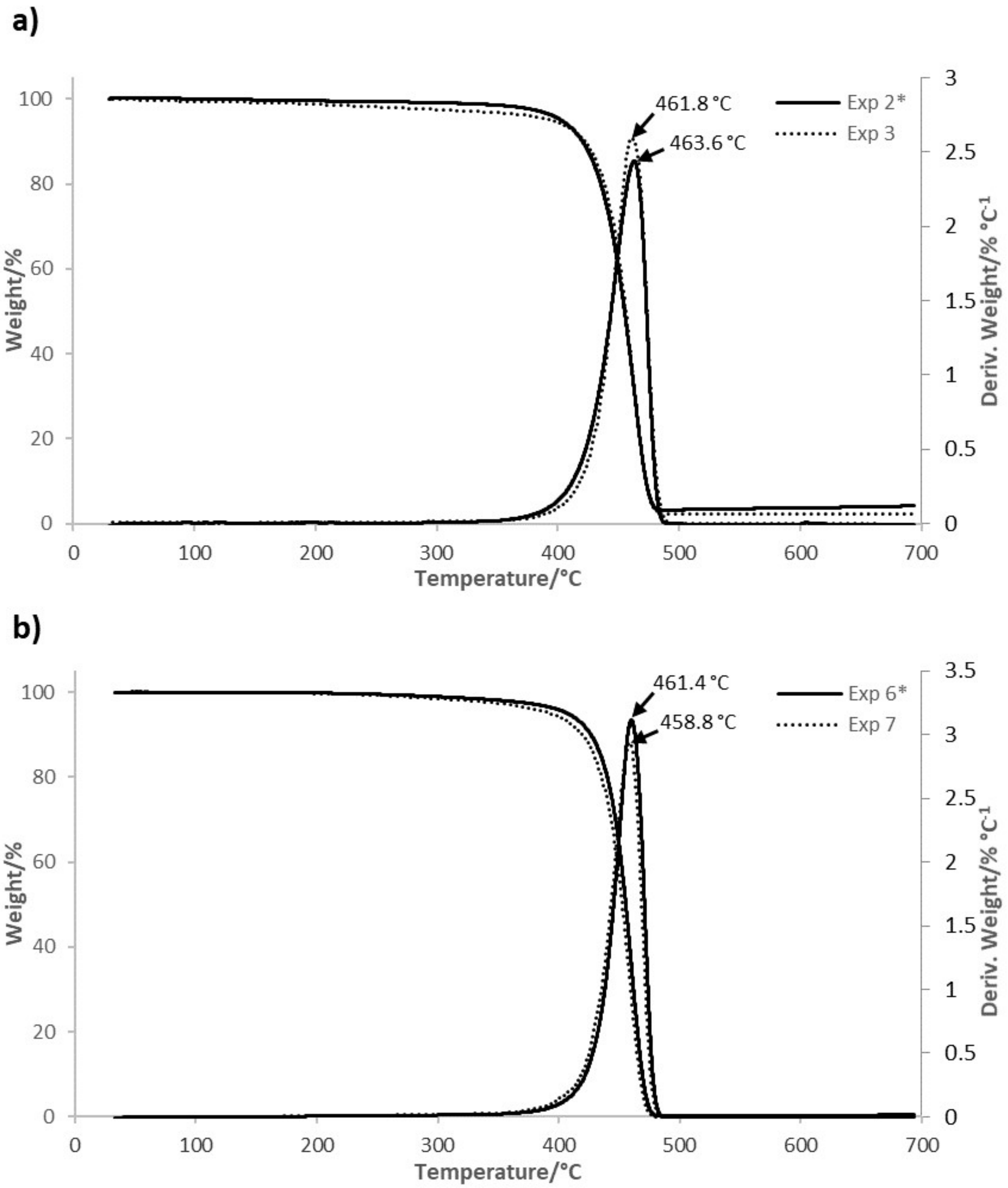

Figure S2. TGA thermograms of the copolymers obtained with a) metallocene and b) Ziegler-Natta catalyst
